# Elderly Friendliness of Hospitals in the Ernakulam District of Kerala, India: A Cross-Sectional Study

**DOI:** 10.7759/cureus.109098

**Published:** 2026-05-18

**Authors:** Aparna Vasudev, Chitra Tomy, Harsha Lais, Gurleen Kaur I Anand

**Affiliations:** 1 Public Health, Amrita Institute of Medical Sciences and Research Centre, Amrita Vishwa Vidyapeetham, Kochi, IND; 2 Ayurveda, Isha Health Solutions, Coimbatore, IND; 3 Community Medicine, Amrita Institute of Medical Sciences and Research Centre, Amrita Vishwa Vidyapeetham, Kochi, IND; 4 Community Medicine, Sree Gokulam Medical College and Research Foundation, Nellanad, IND; 5 Public Health Dentistry, Amrita School of Dentistry, Amrita Vishwa Vidyapeetham, Kochi, IND

**Keywords:** age-friendly policies, delivery of health care, health services accessibility, health services for the aged, hospitals, inpatient care, older adults, quality of health care

## Abstract

Background: An aging population is a major demographic transition with significant implications for healthcare systems, particularly in low- and middle-income countries. Ensuring age-friendly healthcare services is essential to meet the complex needs of the elderly population.

Objective: The objective of this study was to assess the elderly friendliness of government and private hospitals in Ernakulam district, Kerala, India.

Methods: A cross-sectional study was conducted among 70 hospitals (34 government and 36 private) with inpatient facilities in Ernakulam district. A structured checklist was used to assess six domains: accessibility, medical care services, physical environment, inpatient services, admission and billing, and spiritual environment. Data were collected through interviews with administrative personnel and direct observation and analyzed using descriptive and inferential statistical methods.

Results: The mean elderly friendliness score was 27.36 ± 4.78. Overall, 31 (44.3%) hospitals were classified as elderly friendly. Among these, a higher proportion were private hospitals (52.8%) compared with government hospitals (35.3%). The physical environment domain showed the highest performance, whereas accessibility performed the poorest. Private hospitals scored significantly higher in admission and billing (p=0.002) and spiritual environment (p=0.001). Government hospitals showed higher scores in medical care services, but the difference was not statistically significant (p=0.057). No significant difference was observed in overall elderly-friendliness scores between the two sectors (p=0.369).

Conclusion: Less than half of the hospitals in the study were elderly friendly, with major gaps in accessibility infrastructure, geriatric care services, and safety features such as grab rails in toilets. Strengthening hospital accessibility, improving geriatric training among healthcare personnel, and implementing standardized age-friendly policies are required to improve elderly healthcare services.

## Introduction

Population ageing is one of the most significant demographic transitions of the 21st century, driven by declining fertility rates and increasing life expectancy. This transition has profound implications for healthcare systems, particularly in low- and middle-income countries (LMICs), where health services are often not adequately designed to address the complex and chronic needs of older adults [1]. India is experiencing a rapid increase in its elderly population, with individuals aged 60 years and above increasing from 5.6% in 1961 to 8.6% in 2011 [2]. Estimates from the United Nations Population Fund (UNFPA) India Ageing Report 2023 indicate that the proportion of older persons in India was approximately 10.1% in 2021 and is projected to rise to around 15% by 2036 and exceed 20% by 2050, reflecting a rapid demographic transition [3]. Kerala is among the most rapidly ageing states in India, with approximately 13% of its population aged 60 years and above [4].

Older adults frequently face multiple barriers to accessing healthcare, including financial constraints, reduced mobility, and inadequate age-sensitive services. Existing healthcare systems, which are predominantly oriented toward acute care, are often insufficient to address the long-term and multifaceted needs of the elderly. Furthermore, social changes such as nuclearization of families and reduced informal caregiving have intensified the vulnerability of this population [5]. 

Recognizing these challenges, the World Health Organization (WHO) has emphasized the concept of healthy ageing and declared 2021-2030 as the Decade of Healthy Ageing, aiming to improve the well-being and functional ability of older people [6]. In line with this, the WHO introduced the Age-Friendly Primary Health Care Centres Toolkit to provide guidance on adapting healthcare infrastructure and service delivery to improve accessibility, safety, and quality of care for older adults [7]. Several studies have assessed the elderly friendliness of hospitals using the WHO toolkit, and frameworks based on these principles have also been developed in different settings [8,9].

Initiatives such as the National Programme for Health Care of the Elderly (NPHCE) have been introduced in India to strengthen geriatric care services [10]. However, evidence on the elderly friendliness of hospitals in India, particularly in Kerala, remains limited, with few studies comparing government and private sector facilities. Therefore, this study was conducted to assess the elderly friendliness of government and private hospitals in Ernakulam district, Kerala.

## Materials and methods

Study setting and study population

A cross-sectional study was conducted in public and private healthcare facilities with inpatient services in Ernakulam district, Kerala, India, between March 2019 and December 2020. All government Community Health Centres, Taluk Hospitals, District Hospitals, and General Hospitals were included as these facilities represent the full spectrum of secondary and tertiary care public sector hospitals providing inpatient services. Private hospitals with at least one qualified allopathic physician and a minimum of 10 inpatient beds were included to ensure inclusion of functional inpatient care hospitals and to exclude small clinics or single-doctor facilities that do not provide structured inpatient services or comparable hospital-level infrastructure. Facilities providing only specialist services, such as eye, ENT, and dental services, were excluded.

Sample size and sampling technique

The sample size was calculated based on a study by Ahmadi et al. conducted in hospitals in Iran, which reported a 69% prevalence of grab rails in toilets [8]. Using this prevalence (p = 0.69) and assuming a 95% confidence level with 20% allowable error, the minimum sample size was estimated using the formula $n = \frac{Z^2pq}{d^2}$ and was calculated to be 60 hospitals. To improve representativeness, a total of 70 hospitals (government and private) were included in the study.

The list of all government and private hospitals in Ernakulam district was obtained from the District Medical Office (DMO), Ernakulam, which served as the sampling frame. Hospitals meeting the predefined inclusion criteria were selected for the study. All 34 government hospitals that met the inclusion criteria were included after obtaining permission from the District Medical Officer, Ernakulam, Kerala.

**Figure 1:**
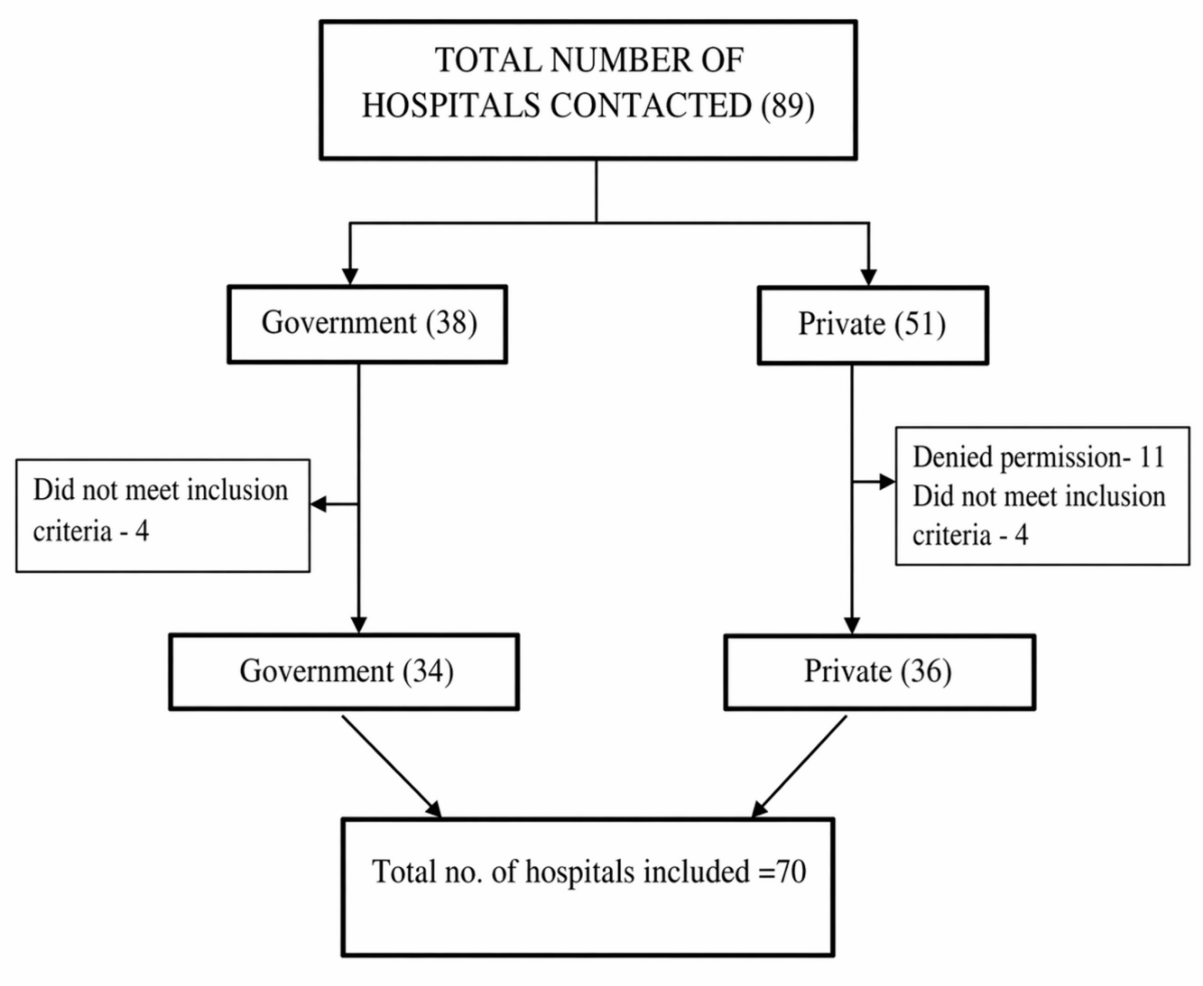
Flowchart of hospital selection and inclusion process

Private hospitals with inpatient facilities were screened based on the inclusion criteria. Hospitals that did not meet the inclusion criteria or did not provide consent were excluded from the study. Eligible private hospitals were included after obtaining permission from the respective hospital administrators. The sampling process is illustrated in Figure 1.

Study tool

A checklist developed and validated by Rashmi et al. in Bangalore, based on the WHO Age-friendly Primary Health Care Centers Toolkit, was used for the study [11]. This was an open-access article distributed under the terms of the Creative Commons Attribution-Non Commercial-Share Alike 3.0 License. The checklist comprised six domains: accessibility, medical care services, physical environment, inpatient services, admission and billing, and spiritual environment.

The final checklist comprised 52 items distributed across the six domains as follows: accessibility (four items), medical care services (13 items), physical environment (26 items), inpatient services (four items), admission and billing (four items), and spiritual environment (one item).

Data collection

After obtaining permission from the concerned authorities, data were collected between March 2019 and December 2020 by the principal investigator, who was trained in the application of the validated checklist to ensure consistency across all study sites. Information on hospital services was obtained through interviews with hospital administrative personnel using a structured checklist, while infrastructural components and facilities were assessed through direct observation. Data were recorded in printed data collection forms during hospital visits and subsequently entered into Microsoft Excel (Microsoft Corporation, Redmond, Washington, United States).

Scoring of the assessment tool

Each checklist item was scored as 1 (“yes”) or 0 (“no”), with a maximum possible total score of 52. The total score was obtained by summing all 52 items. The median score (27) was used as the cut-off value for classification of hospitals into poor elderly-friendliness (<27) and good elderly-friendliness (≥27). Domain-wise scores were computed by summing item scores within each respective domain. Operational definitions and assessment criteria for checklist items were based on the original validated tool by Rashmi et al. [11] and the WHO Age-Friendly Primary Health Care Centers Toolkit [6].

Statistical analysis

Data were analyzed using IBM SPSS Statistics for Windows, Version 20.0 (IBM Corp., Armonk, New York, United States). Descriptive statistics such as frequency and percentage, and mean and standard deviation (SD) were used to summarize the data. The normality of continuous variables was assessed using the Kolmogorov-Smirnov test. Based on the results, appropriate parametric or non-parametric statistical tests, including the independent t-test and Mann-Whitney U test, were used to compare the elderly-friendliness between government and private hospitals. A p-value <0.05 was considered statistically significant.

Ethical approval

The study received ethical approval from the Institutional Review Board of Amrita Institute of Medical Sciences, Kochi (IRB-AIMS-2019-040). Permission was obtained from the District Medical Officer, Ernakulam, and the concerned authorities of participating private hospitals prior to data collection.

## Results

A total of 70 hospitals in Ernakulam District, Kerala, were evaluated for elderly friendliness. The study included government and private hospitals across different levels of care, comprising community health centres, taluk hospitals, taluk headquarters hospitals, general hospitals, district hospitals, and private hospitals. Among the hospitals, 36 (51.4%) were private hospitals and 34 (48.6%) were government hospitals. Of the hospitals, 45 (64.3%) were located in urban areas, and 25 (35.7%) were in rural areas. The majority of hospitals had a bed strength of ≤50 beds, 48 (68.6%), followed by 14 (20%) with 51-300 beds and eight (11.4%) with more than 300 beds. Government hospitals were predominantly located in rural areas, whereas private hospitals were mainly located in urban areas. Government hospitals were further categorized based on their hierarchy, with community health centers accounting for 20 (58.8%), followed by taluk hospitals (n=6, 17.6%), taluk headquarters hospitals (n=5, 14.7%), general hospitals (n=2, 5.9%), and the district hospital (n=1, 2.9%). Out of a maximum possible score of 52 on the elderly-friendly hospital assessment tool, the mean total score was 27.36 ± 4.78. The distribution of scores across each domain is presented in Table 1.

**Table 1:** Distribution of total and domain-wise scores SD: standard deviation

Domain	Mean	SD	Maximum possible score
Total score	27.36	4.78	52
Accessibility	1.77	0.61	4
Medical care services	5.70	2.09	13
Physical environment	17.82	2.41	26
Inpatient services	1.75	0.84	4
Admission and billing	0.41	0.84	4
Spiritual environment	0.25	0.44	1

Hospitals that scored at or above the median were considered to have good elderly friendliness, while those scoring below the median were considered to have poor elderly friendliness. The results showed that 31 (44.3%) hospitals had good elderly friendliness. Among hospital types, 12 (35.3%) government hospitals and 19 (52.8%) private hospitals had good elderly friendliness scores. Based on the domain-wise classification, the highest proportion of hospitals with good elderly friendliness was observed in the physical environment domain, with 24 (34.3%) hospitals, followed by medical care services with 21 (30%), inpatient services with 11 (15.7%), and accessibility with three (4.3%), as shown in Table 2.

**Table 2:** Classification of hospitals according to domain-wise scores and overall elderly-friendliness

Domain	Scores	Hospitals, n (%)
Accessibility	<2	67 (95.7)
	≥2	3 (4.3)
Medical care services	<6	49 (70.0)
	≥6	21 (30.0)
Physical environment	<18	46 (65.7)
	≥18	24 (34.3)
Inpatient services	<2	59 (84.3)
	≥2	11 (15.7)
Elderly friendliness	<27 (Poor)	39 (55.7)
	≥27 (Good)	31 (44.3)

Accessibility to the hospital

Most hospitals were located along the main road (n=57, 81.4%) and near a bus stop or railway station (n=64, 91.4%). However, a separate parking area was available in only two private hospitals, and a separate entrance for older adults was available in only one private hospital.

Medical care services at the hospital

Medical care services varied across hospitals. High proportions of hospitals reported explaining prescribed drugs (n=69, 98.6%), kind and respectful staff (n=70, 100%), and home health services for senior citizens (n=53, 75.7%). Only 11 (15.7%) hospitals had a geriatric clinic, and 23 (32.9%) had staff trained in geriatric care.

Government hospitals showed higher availability in several indicators, including priority service in all service sections and home health services. Among the 41 hospitals that provided priority in pharmacy services, eight provided priorities based on the condition or severity of the patient rather than age, as shown in Table 3.

**Table 3:** Availability of medical care services in government and private hospitals (N=70)

Medical Care Services	Government (n=34), n (%)	Private (n=36), n (%)	Total, n (%)
Priority in pharmacy	22 (64.7)	19 (52.8)	41 (58.6)
Availability of volunteers	11 (32.4)	23 (63.9)	34 (48.6)
Priority in all service sections	25 (73.5)	14 (38.9)	39 (55.7)
Explanation of prescribed drugs	34 (100)	35 (97.2)	69 (98.6)
Separate queue at all counters	11 (32.4)	5 (13.9)	16 (22.9)
Kind, respectful, and patient staff	34 (100)	36 (100)	70 (100.0)
Concession for senior citizens	2 (5.9)	12 (33.3)	14 (20)
Home health service for senior citizens	34 (100)	19 (52.8)	53 (75.7)
Reminders for appointments	2 (5.9)	2 (5.6)	4 (5.7)
Group insurance schemes for senior citizens	10 (29.4)	12 (33.3)	22 (31.4)
Staff trained in geriatric medicine	15 (44.1)	8 (22.2)	23 (32.9)
Separate reception counter for seniors	1 (2.9)	2 (5.6)	3 (4.3)
Geriatric clinic present	6 (17.6)	5 (13.9)	11 (15.7)

Physical environment at the hospital

Physical environment features were generally well-maintained across hospitals. Most hospitals had wide doors, good lighting, railings for staircases, toilets near important patient care areas, western toilets, and signboards in key areas. Elevators were considerably more common in private hospitals than in government hospitals. However, elderly friendly supportive features such as grab rails in toilets, alarms in toilets, and toilet doors opening both ways were limited. The distribution of physical environment features is presented in Table 4.

**Table 4:** Availability of physical environment features in hospitals (N=70)

Indicator	Government Hospitals (n=34), n (%)	Private Hospitals (n=36), n (%)	Total, n (%)
Public telephones available in all important areas	6 (17.6)	7 (19.4)	13 (18.6)
Wide doors	34 (100)	35 (97.2)	69 (98.6)
Good lighting	34 (100)	35 (97.2)	69 (98.6)
Elevators to every floor	3 (8.8)	28 (77.8)	31 (44.3)
Non-slippery floors	22 (64.7)	31 (86.1)	53 (75.7)
Ramps present	27 (79.4)	28 (77.8)	55 (78.6)
Railings for staircases	31 (91.2)	34 (94.4)	65 (92.9)
Well-organized furniture & fittings	31 (91.2)	33 (91.7)	64 (91.4)
Wide & spacious elevators & corridors	26 (76.5)	30 (83.3)	56 (80.0)
Eating outlet within hospital	15 (44.1)	31 (86.1)	46 (65.7)
Accessible eating outlet	26 (76.5)	35 (97.2)	61 (87.1)
Entrance			
Steps at the entrance	25 (73.5)	21 (58.3)	46 (65.7)
Steps with railings	11 (32.4)	11 (30.6)	22 (31.4)
Ramp at the entrance	23 (67.6)	17 (47.2)	40 (57.1)
Ramp with railings	12 (35.3)	9 (25)	21 (30.0)
Toilets			
Toilets available in all important areas of the hospital	32 (94.1)	33 (91.7)	65 (92.9)
Presence of alarm in toilets	0 (0.0)	2 (5.6)	2 (2.9)
Non-slippery toilet floors	22 (64.7)	28 (77.8)	50 (71.4)
Grab rails in toilet	10 (29.4)	9 (25.0)	19 (27.1)
Western closet in every toilet	33 (97.1)	35 (97.2)	68 (97.1)
Toilet doors open both ways	1 (2.9)	3 (8.3)	4 (5.7)
Availability of escort for senior citizens to use toilet	8 (34.0)	6 (16.7)	14 (20.0)
Signboards			
Signboards put up in all important areas	34 (100)	34 (94.4)	68 (97.1)
Big lettering	31 (91.2)	31 (86.1)	62 (88.6)
Bold lettering	33 (97.1)	30 (83.3)	63 (90.0)
Lettering in local language	33 (97.1)	21 (58.3)	54 (77.1)

Inpatient services at the hospital

Separate wards for senior citizens were available in only seven hospitals. Caregivers were allowed to stay with older adults in 64 (91.4%) hospitals. Caregivers were made available on demand in 10 (14.3%) hospitals. Some hospitals had associations with agencies providing home nurses and made them available to older adults in need. Only nine private hospitals and one government hospital had this provision. More than half of the hospitals (n=42, 60%) had recreation facilities for older adults.

Admission and billing services, and spiritual environment

Priority for admission was available in 13 private hospitals compared with two government hospitals. Priority at the billing counter was reported in seven private hospitals compared with one government hospital. Separate billing counters were present in three (8.3%) private hospitals, and an equal number reported preparing bills on the previous day. Prayer halls were absent in government hospitals, but were present in 18 (50%) private hospitals. Only 21 (30%) hospitals had a written age-friendly policy, and health information on the NPHCE was available in only four (5.7%) hospitals. Screening for diabetes and hypertension was available in all hospitals, whereas services for geriatric conditions, such as falls (n=45, 64.3%), depression (n=54, 77.1%), and memory loss (n=52, 74.3%) were less consistently available.

The mean total score was similar in private hospitals (27.86 ± 5.81) and government hospitals (26.82 ± 3.41), with no statistically significant difference in overall elderly friendliness (t = 0.905, p = 0.369). Similarly, no significant difference was observed in physical environment scores between government and private hospitals (t = 0.564, p = 0.151), as shown in Table 5.

**Table 5:** Comparison of mean scores between government and private hospitals Independent t-test used for comparison; p < 0.05 considered statistically significant.

Variable	Government (Mean ± SD)	Private (Mean ± SD)	t-value	p-value
Total score	26.82 ± 3.41	27.86 ± 5.81	0.905	0.369
Physical environment	17.26 ± 2.35	17.64 ± 3.12	0.564	0.151

The mean rank for admission and billing was higher in private hospitals (40.94) than in government hospitals (29.74), and this difference was statistically significant (Mann-Whitney U = 416.0, p = 0.002). The mean rank for spiritual environment was also higher in private hospitals (44.0) than in government hospitals (26.5), and this difference was statistically significant (Mann-Whitney U = 306.0, p = 0.001). No significant differences were observed for accessibility, inpatient services, or medical care services, as shown in Table 6.

**Table 6:** Comparison of mean ranks for domains across government and private healthcare institutions *Mann–Whitney U test used for comparison; p < 0.05 considered statistically significant.

Domains	Government Hospitals, mean rank	Private Hospitals, mean rank	Mann-Whitney U value	p-value
Accessibility	32.01	38.79	493.5	0.069
Medical care services	40.19	31.07	452.5	0.057
Inpatient services	34.81	36.15	588.5	0.766
Admission and billing	29.74	40.94	416.0	0.002*
Spiritual environment	26.50	44.00	306.0	0.001*

## Discussion

This study provides important insights into elderly friendly healthcare services in a high-ageing setting in India. Nearly half of the hospitals demonstrated acceptable levels of elderly friendliness, with the physical environment showing better performance than other domains. However, substantial gaps persisted across key domains.

In India, elderly friendly healthcare practices are supported through Indian Public Health Standards (IPHS) [12], National Accreditation Board for Hospitals and Healthcare Providers (NABH) standards [13], and the NPHCE [10]. These recommend barrier-free access, ramps, railings, accessible toilets, separate queues, geriatric clinics, and priority services for older adults. However, the present study identified gaps in the implementation of several of these measures, particularly in government hospitals, including limited availability of grab rails, separate billing counters, and dedicated geriatric facilities.

Accessibility emerged as the weakest domain, reflecting inadequate prioritization of elderly specific infrastructure in hospital design. This finding is consistent with studies from other LMICs, where structural barriers continue to limit healthcare access for older adults. A study done in Iran by Ahmadi et al. showed that none of the hospitals had separate parking spaces for senior citizens [8]. Similarly, our study found limited provision for elderly specific parking or entrances. Rashmi et al. reported that 45% of elderly participants in Bangalore preferred hospitals located near public transport facilities [11]. In the present study, most hospitals (91.4%) were located near a bus stop or railway station; however, frequent transport services were still limited in some rural areas.

Private hospitals generally performed higher across domains, but government hospitals performed better in medical care services, consistent with Kerala accreditation standards requiring separate queues for the elderly at district and general hospitals [14]. Previous studies confirm that most elderly prefer priority at service counters, and long waiting times are a major challenge [11,15]. Although staff in all hospitals were reported as kind and respectful, this may reflect social desirability bias. Only a minority of hospitals had trained geriatric personnel, indicating the need for strengthening geriatric capacity building within the healthcare workforce.

Although affordability was not directly assessed in the present study, previous studies from Nepal and India have shown that older adults often face financial barriers in accessing healthcare [15,16]. Family support may also decline over repeated episodes of illness, increasing the financial burden on older adults [17]. Government hospitals in the present study consistently provided home health services under palliative care, whereas private hospitals lagged behind, despite evidence that many older adults prefer home-based care [18].

Physical environment was the strongest domain, particularly ramps, railings, and non-slippery toilets. Evidence suggests that such modifications help prevent falls and related injuries [19,20]. However, only about one-fourth of the hospitals had grab rails in toilets, even though falls are most common in bathrooms among the elderly [15]. The absence of critical safety features highlights gaps in the full implementation of age-friendly standards.

Few hospitals had dedicated wards, though caregiver presence was allowed in most. Recreation facilities were more common but not universal. Elderly patients often expect simple amenities like a TV to pass the time [15]. Admission and billing systems in private hospitals were more elderly friendly, offering priority counters and faster billing. In contrast, most government hospitals lacked such provisions, similar to findings in Iran [8].

Spiritual care, an often-overlooked aspect of holistic healthcare, was inadequately addressed in government hospitals. Considering its role in improving the well-being of elderly patients, integrating spiritual care into healthcare delivery warrants attention [21]. Incorporating dedicated prayer spaces within hospitals may help address the spiritual needs of older adults.

Organizational support and preventive services were also suboptimal. Only a minority of hospitals had a written age-friendly policy, and awareness regarding national geriatric health programs was limited. Although screening for common non-communicable diseases was widely available, services targeting geriatric-specific conditions were less consistent, indicating a gap in comprehensive geriatric care.

Overall, nearly half of the hospitals were elderly friendly. However, important gaps remained in accessibility-related infrastructure, dedicated geriatric services, trained geriatric personnel, toilet safety features, and elderly supportive admission and billing services. While private hospitals performed better in infrastructure-related domains, government hospitals showed better performance in selected medical care services for older adults. The findings highlight the need for improved geriatric-friendly infrastructure, staff training, and strengthening of elderly-focused healthcare services across hospitals.

 Limitations

The cross-sectional design of the study limits the ability to establish causal relationships between hospital characteristics and elderly friendliness. The scoring system assigned equal weight to all checklist items, which may not reflect the relative importance of individual components. Additionally, the study was conducted in a single district, which may limit the generalizability of the findings. Responses obtained from hospital administrative personnel and observations may be subject to social desirability and observer bias. The study has limited analytical depth due to its primarily descriptive cross-sectional design.

Recommendations

A structured, elderly friendly hospital framework should be developed and implemented across healthcare facilities. Hospitals should strengthen elderly friendly infrastructure by ensuring accessible entrances, ramps with railings, grab rails in toilets, non-slippery flooring, elevators, and clear signboards. Provision of separate queues and priority services for older adults should be encouraged. In addition, geriatric care services should be strengthened through staff training, development of written elderly friendly policies, and improved implementation of elderly friendly components within existing accreditation and quality improvement systems. Greater awareness and effective implementation of the NPHCE should be promoted across healthcare facilities

## Conclusions

Less than half of the hospitals in Ernakulam district were elderly friendly, with significant gaps across multiple domains, including accessibility, medical care services, physical environment, inpatient services, and admission and billing systems. This highlights the need for the structured implementation of age-friendly hospital standards and integration of geriatric care principles into routine healthcare delivery in view of Kerala’s ageing population.
